# Quality of life and sexual function after TVT surgery versus Bulkamid injection for primary stress urinary incontinence: 1 year results from a randomized clinical trial

**DOI:** 10.1007/s00192-020-04618-5

**Published:** 2020-12-04

**Authors:** Anna-Maija Itkonen Freitas, Tomi S. Mikkola, Päivi Rahkola-Soisalo, Sari Tulokas, Maarit Mentula

**Affiliations:** 1 Department of Obstetrics and Gynecology, Helsinki University Hospital, Helsinki University, PO BOX 140, 00029 HUS, Helsinki, Finland; 2 Department of Obstetrics and Gynecology, Helsinki University Hospital and Folkhälsan Research Center Biomedicum, Helsinki University, Helsinki, Finland; 3 Doctoral Programme in Clinical Research, Helsinki University, Helsinki, Finland

**Keywords:** Mesh sling, Bulking, Polyacrylamide hydrogel

## Abstract

**Introduction and hypothesis:**

To assess changes in quality of life (QoL) and sexual function outcomes at 1 year after tension-free vaginal tape (TVT) versus polyacrylamide hydrogel injection (PAHG).

**Methods:**

In a randomized trial comparing TVT (*n* = 111) and PAHG (*n* = 113) treatments of stress urinary incontinence (SUI), we compared urinary incontinence and health-related QoL using the Urogenital Distress Inventory (UDI-6), Incontinence Impact Questionnaire, Short Form (IIQ-7), Pelvic Organ Prolapse/Urinary Incontinence Sexual Questionnaire (PISQ-12) and RAND-36 Item Health Survey (RAND-36) at baseline and 1 year.

**Results:**

UDI-6 and IIQ-7 showed improved incontinence-related QoL (*p* = 0.001) from baseline in both groups except for difficulty emptying the bladder and pain/discomfort. At 1 year, TVT patients experienced less urinary symptom-related distress compared to PAHG (*p* < 0.001). Sexual function improved in both groups (*p* < 0.001 for TVT and *p* = 0.01 for PAHG) with higher scores for the physical section subscale (*p* < 0.001) for TVT. Health-related QoL (RAND-36) improved from baseline in both groups in physical and social functioning (*p* < 0.001) with better outcome in the TVT group for physical functioning (*p* < 0.001). Increase in pain from baseline (*p* = 0.02) was detected for TVT, but not for PAHG. However, there was no difference between the groups (*p* = 0.78).

**Conclusions:**

In primary SUI, TVT and PAHG treatments both improved QoL and sexual function at 1 year. However, incontinence and health-related QoL scores were better in the TVT group. More pain compared to the baseline was reported after TVT, although there was no difference between groups. Clinical significance needs to be evaluated in long-term follow-up.

## Introduction

Urinary incontinence affects up to 46% of adult women [1], markedly reducing the quality of life (QoL) [2], and even minimal incontinence is associated with a significant decline in QoL [3]. The majority of female urinary incontinence is associated with exertion, sneezing or coughing, i.e., stress urinary incontinence (SUI) [4]. In addition to a negative impact on overall QoL, sexual dysfunction is also commonly reported in women with SUI [5].

The first-line treatment of SUI, pelvic floor muscle training, increases QoL [6]. However, many women require invasive treatments, and the most common surgical option for SUI is midurethral sling surgery with retropubic tension-free vaginal mesh tape (TVT), which has a high success rate [7]. However, mesh complications, such as erosion, obstruction and pain [8], have raised concerns, and in England the new NICE Guidelines advise that midurethral slings only be considered if other procedures are not suitable [9]. Furthermore, studies have shown that women are prepared to trade efficacy for safety by preferring a minimally invasive procedure [10, 11].

An alternative and minimally invasive treatment option for SUI is bulking. Transurethral polyacrylamide hydrogel (PAHG, Bulkamid®) is a homogeneous gel that has been used for more than 10 years to treat SUI in women, and it has been proven to be a safe intervention [12–14]. Bulking has been typically used as salvage treatment in elderly patients. However, PAHG use is also gaining popularity for primary SUI in younger women. However, little is known about QoL or sexual function following bulking treatments [15].

Several studies have shown improved QoL [7, 16], and improved sexual function after midurethral sling placement [17–19]. Previous studies with PAHG used as a salvage treatment show improved QoL [20–23] and improved sexual function [20]. However, among primary SUI patients the effect of PAHG in QoL has not been reported.

We conducted a randomized trial comparing TVT and PAHG treatments in women with primary SUI [24]. The aim of the present study was to assess QoL and sexual function outcomes at 1 year following TVT or PAHG injections using both urinary incontinence-specific and general health-related quality of life (HRQoL) questionnaires.

## Materials and methods

Two hundred twenty-four women suffering from SUI with inadequate response to pelvic floor muscle training were included in this trial conducted at Helsinki University Hospital, Helsinki, Finland, as reported before [24]. Briefly, the women were referred to our clinic for SUI treatment and after written consent randomized 1:1 to TVT (*n* = 111) or PAHG (*n* = 113) treatments. Both procedures were performed in an outpatient setting using local anesthesia. The TVT (TVT-Exact®, Gynecare, Ethicon, Johnson & Johnson, USA) was inserted as originally described [25]. PAHG injections (Bulkamid®, Contura, Denmark) were injected under endoscopic control 1.5 cm from the vesico-urethral junction to four sites with hydrogel cushions meeting at midline [19]. Six women in the TVT group and five women in the PAHG group withdrew their consent after randomization, one woman accidentally received two randomization envelopes, and four women were lost to follow-up. Two hundred twelve women (TVT *n* = 104 and PAHG *n* = 108) underwent treatment as randomized and were included for this 1-year analysis.

Patients gave written informed consent before enrollment. The study was approved by the Helsinki University Ethics Committee and registered in Clinical Trials, NCT02538991.

Urinary incontinence-related QoL outcomes were assessed using the Urogenital Distress Inventory (UDI-6), Incontinence Impact Questionnaire Short Form (IIQ-7) and Pelvic Organ Prolapse/Urinary Incontinence Sexual Questionnaire (PISQ-12). General HRQoL was assessed using the RAND-36 Item Health Survey (RAND-36) at baseline and at 1-year follow-up.

As incontinence-specific questionnaires, we used UDI-6 and IIQ-7 to assess symptom distress and the impact of incontinence on daily life with the total score ranging from 0 to 100. Higher points indicate more distress. For the UDI-6 and IIQ-7 minimally important difference (MID), the smallest change in a questionnaire score that is found to correlate with meaningful symptom improvement has not been defined but ≥ 75% improvement in UDI-6 and IIQ-7 has been used while defining MID for ICIQ-UI-SF [26]. Therefore, to try to evaluate clinical difference and not only statistical difference alone, we calculated in how many women ≥ 75% improvement was reached.

The impact of SUI on a patient’s sexual life during the last 6 months was measured with the PISQ-12 questionnaire [27]. Three subscales can be identified: behavior-emotional, physical and partner related. The maximum score is 48 with higher points indicating better sexual satisfaction.

To measure HRQoL we used the RAND-36 questionnaire [28] including eight multi-item dimensions: general health, physical functioning, mental health, social functioning, vitality, pain, and physical and emotional role functioning. Higher scores indicate better HRQoL.

The study was conducted as a noninferiority trial based on rates of patient satisfaction with treatment [24] and QoL, and sexual function analyses were secondary outcomes. All statistical analyses were performed using IBM SPSS Statistics, version 24.0 (SPSS Inc., Chicago IL, USA). Data on the questionnaires are presented as means ± SD. Student’s *t*-test for paired samples was used to test changes in outcome measures within the groups before and 1 year after the treatment. Student’s *t*-test for independent samples was used to analyze possible differences between the two treatment groups. Data are presented on an intention-to-treat basis but we also performed per protocol analyses to confirm that the results did not significantly differ. *P* < 0.05 was considered significant for all analyses.

## Results

Baseline characteristics were similar between the groups (Table 1). The mean age of the participating women was 51.0 years; 43.4% were postmenopausal; the mean BMI was 25.0 and mean parity 2.1.

**Table 1:** Demographics of the 212 women undergoing TVT or PAHG treatment. Intention-to-treat data^*^

	TVT group *n* = 104	PAHG group *n* = 108
Age, mean, ± SD (range)	50.4 ± 10.4 (32.0–78.0)	51.5 ± 11.0 (31.0–80.0)
Postmenopausal	44 (42.3)	48 (44.4)
Systemic HRT	20 (19.2)	25 (23.1)
Vaginal estrogen	29 (27.9)	35 (32.4)
BMI (kg/m^2^), mean ± SD (range)	24.5 ± 3,5 (16.1–34.9)	24.8 ± 3.6 (18.9–34.2)
Smoking	14 (13.5)	10 (9.3)
Socioeconomic status		
Working	83 (79.8)	86 (79.6)
White and blue collar workers	85 (81.7)	87 (80.5)
Others	19 (18.3)	21(19.5)
Parity/delivery, mean ± SD (range)	2.1 ± 1.0 (0–5)	2.1 ± 0.9 (0–6)
0	7 (6.7)	4 (3.7)
Vaginal deliveries	93 (89.4)	101 (93.5)
Cesarean section only	4 (3.8)	3 (2.8)
Duration of incontinence		
> 1 and < 2 years	1 (1.0)	4 (3.7)
2 to 5 years	64 (61.5)	65 (60.2)
> 5 years	39 (37.5)	39 (36.1)
Distress from incontinence (VAS 0–10)**, mean ± SD (range)	8.0 ± 1.4 (4–10)	8.1 ± 1.4 (4–10)

TVT, tension-free vaginal tape; PAHG, polyacrylamide hydrogel; SD, standard deviation; HRT, hormone replacement therapy; BMI, body mass index; VAS, visual analogue scale

^*^Data are presented as number of patients (%) unless otherwise stated

^**^n = 1 for missing PAHG group data

**Fig. 1:**
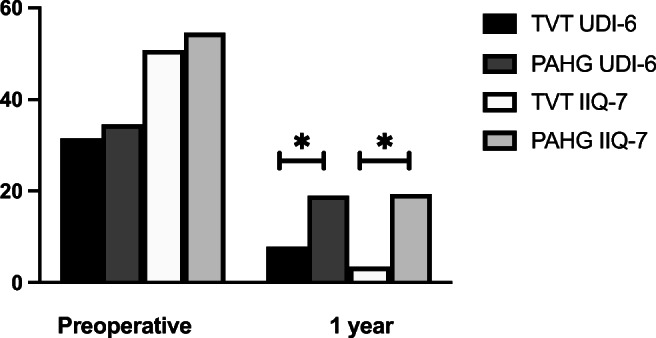
Mean scores for UDI-6 and IIQ-7, * = *p* < 0.001

The UDI-6 and IIQ-7 questionnaires were filled adequately at baseline by 101 (97.1%) women in the TVT group and 107 (99.1%) in the PAHG group and after 1 year by 103 (99.0%) and 108 (100.0%) women, respectively. The baseline scores showed no differences between the groups, and most distress was reported with stress-related leakage and impact of incontinence on physical activities (Tables 2, 3 and Fig. 1). At 1 year in UDI-6 and IIQ-7, the total score and individual questions showed significant improvement from baseline in both groups except for UDI-6 questions 5–6 about difficulty emptying the bladder and pain or discomfort (Tables 2, 3 and Fig. 1). Total scores of UDI-6 and IIQ-7 indicate less urinary symptom-related distress among TVT patients compared to PAHG (*p* < 0.001) (Fig. 1). All individual UDI-6 and IIQ-7 questions, except for difficulty emptying the bladder and pain, also show better improvement in the TVT group compared to the PAHG group after 1 year (Table 2). Women in the PAHG group scored highest for stress-related leakage and impact on physical activities. In the TVT group 62 (59.6%) and in the PAHG group 21 (19.4%) reached the ≥ 75% improvement in UDI-6 (*p* < 0.001) and for IIQ-7 93 (89.4%) and 52 (48.1%), respectively (*p* < 0.001).

**Table 2:** Results from UDI-6

UDI-6	Baseline TVT *n* = 104	Baseline PAHG *n* = 108	One year TVT, *p* within-group difference	One year PAHG, *p* within-group difference	*p* between-group difference
Frequent urination	0.99 ± 0.92 (95% CI 0.81–1.17)	1.26 ± 0.94 (95% CI 1.07–1.44)	0.46 ± 0.61 (95% CI 0.34–0.58), *p* = < 0.001	0.72 ± 0.79 (95% CI 0.57–0.87), *p* = < 0.001	0.01 (95% CI -0.46–0.75)
Urine leakage urgency	1.01 ± 0.96 (95% CI 0.82–1.20)	1.23 ± 1.04 (95% CI 1.01–1.48)	0.25 ± 0.46 (95% CI 0.16–0.34), *p* = < 0.001	0.72 ± 0.77 (95% CI 0.57–0.87), *p* = < 0.001	< 0.001 (95% CI -0.67–0.19)
Urine leakage stress	2.76 ± 0.47 (95% CI 2.67–2.86)	2.78 ± 0.46 (95% CI 2.69–2.86)	0.15 ± 0.36 (95% CI 0.08–0.22), *p* = < 0.001	1.36 ± 0.96 (95% CI 1.17–1.54), *p* = < 0.001	< 0.001 (95% CI -1.40–0.99)
Dropping	2.06 ± 0.90 (95% CI 0.09–1.89)	2.13 ± 0.78 (95% CI 1.98–2.28)	0.33 ± 0.5 (95% CI 0.23–0.44), *p* = < 0.001	1.02 ± 0.82 (95% CI 0.86–1.18), *p* = < 0.001	< 0.001 (95% CI -0.87–0.49)
Difficulty emptying bladder	0.42 ± 0.68 (95% CI 0.28–0.55)	0.43 ± 0.72 (95% CI 0.29–0.57)	0.50 ± 0.61 (95% CI 0.38–0.63), *p* = 0.29	0.50 ± 0.71 (95% CI 0.37–0.64), *p* = 0.33	0.96 (95% CI -0.18-0.18)
Pain or discomfort	0.32 ± 0.63 (95% CI 0.19–0.44)	0.39 ± 0.60 (95% CI 0.27–0.50)	0.34 ± 0.67 (95% CI 0.21–0.47), *p* = 0.81	0.27 ± 0.56 (95% CI 0.20–0.38), *p* = 0.06	0.42 (95% CI -0.10-0.24)
Total score (max 100)	31.48 ± 11.51 (95% CI 29.21–33.75)	34.57 ± 10.85 (95% CI 32.49–36.65)	7.89 ± 7.65 (95% CI 6.38–9.40), *p* = < 0.001	18.96 ± 13.20 (95% CI 16.43–21.50), *p* = < 0.001	< 0.001 (95% CI -3.37–1.95)

**Table 3:** Results from IIQ-7

IIQ-7	Baseline TVT	Baseline PAHG	One year TVT, *p* within-group difference	One year PAHG, *p* within-group difference	*p* between-group difference
Household chores	1.12 ± 0.80 (95% CI 0.96–1.28)	1.10 ± 0.90 (95% CI 0.93–1.28)	0.05 ± 0.22 (95% CI 0.01–0.09), *p* = < 0.001	0.43 ± 0.69 (95% CI 0.30–0.57), *p* = < 0.001	< 0.001 (95% CI -0.52–0.24)
Physical acitivities	2.68 ± 0.56 (95% CI 2.57–2.80)	2.68 ± 0.58 (95% CI 2.57–2.79)	0.22 ± 0.56 (95% CI 0.11–0.39), *p* = < 0.001	1.17 ± 0.99 (95% CI 0.98–1.36), *p* = < 0.001	< 0.001 (95% CI -1.16–0.72)
Entertainment activities	1.56 ± 1.03 (95% CI 1.35–1.76)	1.57 ± 0.87 (95% CI 1.40–1.74)	0.07 ± 0.30 (95% CI 0.01–0.13), *p* = < 0.001	0.49 ± 0.80 (95% CI 0.34–0.65), *p* = 0.00	< 0.001 (95% CI -0.57–0.24)
Ability to travel	1.17 ± 0.80 (95% CI 0.97–1.36)	1.40 ± 1.04 (95% CI	0.07 ± 0.26 (95% CI 0.02–0.12), *p* = < 0.001	0.42 ± 0.77 (95% CI *p* = < 0.001	< 0.001 (95% CI-0.52–0.19)
Participating social activities	1.34 ± 0.93 (95% CI 1.16–1.53)	1.38 ± 0.86 (95% CI 1.22–1.55)	0.09 ± 0.38 (95% CI 0.02–0.17), *p* = < 0.001	0.39 ± 0.65 (95% CI 0.27–0.52), *p* = < 0.001	< 0.001 (95% CI -0.47–0.17)
Emotional health	0.89 ± 0.90 (95% CI 0.71–1.01)	1.17 ± 0.97 (95% CI 0.98–1.35)	0.08 ± 0.27 (95% CI 0.03–0.13), *p* = < 0.001	0.37 ± 0.68 (95% CI 0.24–0.50), *p* = < 0.001	< 0.001 (95% CI -0.43–0.15)
Feeling frustrated	1.91 ± 0.92 (95% CI 1.73–2.10)	2.11 ± 0.88 (95% CI 1.94–2.28)	0.15 ± 0.44 (95% CI 0.06–0.24), *p* = < 0.001	0.79 ± 0.88 (95% CI 0.62–0.95), *p* = < 0.001	< 0.001 (95% CI 0.82–0.43)
Total score (max 100)	50.79 ± 19.01 (95% CI 47.04–54.54)	54.50 ± 20.05 (95% CI 50.66–58.35)	3.49 ± 8.61 (95% CI 1.79–5.20), *p* = < 0.001	19.34 ± 21.01 (95% CI 15.31–23.37) **p** = < 0.001	< 0.001 (95% CI -4.23–2.38)

Sexual activity did not differ between the groups; 72 (69.2%) women in the TVT group and 71 (65.7%) in the PAHG group were sexually active at baseline and at 1 year 72 (69.2%) and 73 (67.6%), respectively. The mean age of sexually inactive women was higher [57.1 ± 10.6 (37.0–80.0) vs. 48.0 ± 9.4 (31.0–76.0)] and they were more often postmenopausal [47 (69.1%) vs. 44 (30.8%)] compared to sexually active women. Subjective distress VAS from incontinence did not differ between the groups. A total of 64.6% of all the patients remained sexually active, 26.4% remained sexually inactive, 3.8% became sexually active (*n* = 3 in the TVT group and *n* = 5 in the PAHG group), and 3.3% became sexually inactive (*n* = 2 in the TVT group and n = 5 in the PAHG group) with no difference between the treatment groups. Total score points improved in both groups showing improvement in overall sexual satisfaction (*p* < 0.001 for TVT and *p* = 0.01 for PAHG), and there was no difference between the groups (TVT 36.84 ± 5.27 vs. PAHG 34.95 ± 6.33; *p* = 0.05, 95% CI -0.03-3.81). In the subscale analyses, behavior-emotional or partner-related sections showed no significant improvement from baseline to 1 year and no difference between the groups. On the physical subscale there was significant improvement in both groups between baseline and 1 year (*p* < 0.001 for both TVT and PAHG) with higher scores in the TVT group at 1 year (14.69 ± 1.38 in the TVT group vs. 13.22 ± 2.6 in the PAHG group, *p* < 0.001, 95% CI 0.79–2.16). Postoperative dyspareunia remained unchanged in both groups (*p* = 0.38 for TVT and *p* = 0.52 for PAHG), and there was no difference between the groups at 1 year (*p* = 0.70).

**Fig. 2:**
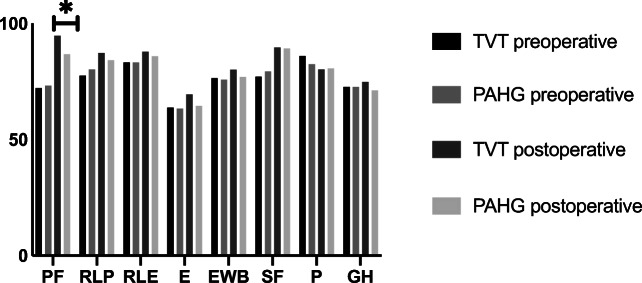
Mean scores for RAND-36, *p < 0.001. PF = physical functioning, RLP = role limitations due to physical health, RLE = role limitations due to emotional problems, E = energy/fatigue, EWB = emotional well-being, SF = social functioning, P = pain, GH = general health

Health-related QoL by RAND-36 showed significant improvement from baseline in physical functioning and social functioning in both groups with better outcome in the TVT group for physical functioning (*p* < 0.001, Fig. 2). In the TVT group, energy (*p* < 0.001) and emotional well-being (*p* = 0.02) improved from baseline without any difference between the groups at 1 year. A lower score indicating more pain at 1 year compared to baseline (86.0 vs. 80.2, *p* = 0.02, 95% CI 0.9–10.4) was detected only in the TVT group. However, there was no significant difference in the comparison between the groups (*p* = 0.78).

## Discussion

Our results show that after 1 year both TVT and PAHG treatments improve urinary incontinence-related QoL, sexual satisfaction, and physical and social functioning of HRQoL. However, the QoL in women with TVT exceeded that of women with PAHG in most of the studied domains. The difference was most evident in the questions on stress urine leakage and physical activities, which correlates well with the better subjective and objective efficacy of TVT compared to PAHG [24]. Interestingly, at 1 year more pain was detected only in the TVT group, although there was no difference between the two treatments.

Our data are in accordance with previous studies indicating improved QoL in UDI-6 and IIQ-7 in up to 5 years of follow-up after midurethral sling surgery [7, 16]. Although less studied and with shorter follow-up, significant improvement in IIQ-7 as well as in another incontinence-specific questionnaire, ICIQ-SF, has also been shown for PAHG [12]. Direct comparison between various studies is challenging, as different validated questionnaires are commonly used to measure incontinence-related QoL. Association between different measures of incontinence severity and QoL also differ [29]. However, in our study evaluation of clinically significant improvement in UDI-6 and IIQ-7 (≥ 75% improvement in scores) showed better improvement in QoL in the TVT group.

Mesh-related complications, including difficulties emptying the bladder and pain, have raised concerns in recent years [8, 30]. In our study, the TVT group showed worsening in the pain dimension of RAND-36 between baseline and 1 year with no change in the PAHG group indicating tape or TVT procedure-related pain increase. However, in incontinence-specific UDI-6 question 6 for pain or discomfort and PISQ-12 question 5 for pain during sexual intercourse, no change was detected from baseline in either group. No change in difficulties emptying the bladder was detected in either group. Since mesh-related complications can emerge years after the initial treatment [8], long-term follow-up is needed to define the clinical significance of the increased pain detected in the TVT group and possible difficulties in bladder emptying.

Women reporting urinary incontinence or lower urinary tract symptoms complain significantly more about sexual dysfunction than the general healthy population without urinary symptoms [31]. In our data, 65–70% of the women reported being sexually active, which is in line with previous reports about female SUI patients of this age [19, 32]. Based on our study, we may not elaborate the specific reasons for non-activity; thus, we do not know whether they were possibly related to SUI. As expected, in sexually inactive women the mean age was higher, and they were more often postmenopausal compared to sexually active women. Sexual activity did not significantly increase after 1 year with no difference between the groups. Thus, it is likely that the reasons for sexual inactivity are multifactorial. This is also supported by our data showing that both the behavioral-emotional and partner-related domains remained unchanged, whereas the physical domain improved in both groups. This increase was also notable in total scores, which is in accordance with previous results after midurethral sling surgery [5, 17, 19, 32]. Data on PAHG are sparse, and only one study reports PAHG treatment’s impact on sexual function with significant improvement in quality of sexual life at 12 months after treatment [20]. Cure of coital incontinence or absence of fear of incontinence during sexual activity seems to correlate well with postoperative sexual satisfaction [5, 17, 19]. This was also detected in our study showing the best improvement in avoiding sexual activity due to fear of incontinence. The women with TVT gained more benefit than the women with PAHG, and this is likely due to better objective cure in the TVT group [24].

The baseline RAND-36 scores for physical functioning were significantly lower than the mean scores of the age-matched Finnish women [28], highlighting the negative impact of urinary incontinence on HRQoL [2, 3]. However, after 1 year women in both groups reached the level of age-matched Finnish women [28]. Both groups showed the most benefit in physical functioning, although women with TVT exceeded that of PAHG. Our data support previous studies showing the positive impact of midurethral slings on improving the QoL of women with SUI [7]. Bulking agents, including PAHG [33], showed a significantly better rating for the domains of general health and role limitations in the King’s Health Questionnaire. Other studies with different questionnaires also show improvement in quality of life after PAHG [21–23]. In our study, significant improvement in the PAHG group was seen in physical and social functioning. However, in physical functioning TVT patients scored higher at 1 year.

Our study has limitations. We studied only TVT and PAHG treatments; thus, our data cannot be extended to different midurethral slings or to other bulking agents. Furthermore, our study on QoL and sexual function was a secondary analysis; thus, in all of the outcomes the power might not have been adequate to show a statistically significant difference. Also, only 65–70% of the study population was sexually active, and we could not determine if the reason for non-activity was possibly SUI related. However, our sexual activity rates are similar to those of previous studies; furthermore, sexual activity did not significantly increase after 1 year. We used comprehensive measures of QoL, the International Continence Society’s recommended questionnaires UDI-6 and IIQ-7 as urinary incontinence specific and PISQ-12 and RAND-36 for sexuality and HRQoL. We acknowledge that choosing other validated questionnaires might have provided additional QoL information.

The strengths of our study include the randomized setting, excellent response rate to the questionnaires, low drop-out rate and comprehensive measures of HRQoL. Moreover, our study compared women having primary SUI undergoing TVT or PAHG, whereas previous studies with PAHG have mainly included women with mixed urinary incontinence or elderly women and women with several previous urinary incontinence surgeries undergoing “salvage procedures” when previous treatments have failed. Our randomized trial setting included standardized questionnaires at exact time points. Furthermore, these data support our previous report of subjective and objective efficacy outcomes [24].

In conclusion, this study shows that in women with primary SUI both TVT and PAHG treatments improved HRQoL and sexual function at 1-year follow-up, but the changes were better with TVT. Although in HRQoL the TVT group reported more pain at 1 year compared to baseline, the difference between the TVT and PAHG groups was not significant. The clinical significance of this finding needs to be evaluated in long-term follow-up.
